# Cutaneous Perfusion Dynamics of the Lower Abdomen in Healthy Normal Weight, Overweight and Obese Women: Methods Development Using Infrared Thermography with Applications for Future Wound Management after Caesarean Section

**DOI:** 10.3390/ijerph20065100

**Published:** 2023-03-14

**Authors:** Charmaine Childs, Harriet Nwaizu, Elizabeth Bullivant, Jon Willmott, Matthew Davies, Karen Ousey, Hora Soltani, Richard Jacques

**Affiliations:** 1 Centre for Applied Health & Social Care Research, Health Research Institute, Sheffield Hallam University, Sheffield S10 2BP, UKe.bullivant@shu.ac.uk (E.B.);; 2 Semiconductor Materials and Devices Research Group, Department of Electronic and Electrical Engineering, University of Sheffield, Sheffield S10 2TN, UK; j.r.willmott@sheffield.ac.uk (J.W.); matt.davies@sheffield.ac.uk (M.D.); 3 Institute of Skin Integrity and Infection Prevention, University of Huddersfield, Huddersfield HD1 3DH, UK; k.j.ousey@hud.ac.uk; 4 Medical Statistic Group, School of Health and Related Research (ScHARR), University of Sheffield, Sheffield S1 4DA, UK; r.jacques@sheffield.ac.uk

**Keywords:** infrared thermography, dynamic, cutaneous, perfusion, wound, complications, caesarean section, body mass index, women, BMI, body habitus, abdomen, personalised medicine

## Abstract

Background: Evidence has shown an association between obesity and an increased risk of wound infection after caesarean section. This study was designed to examine if abdominal subcutaneous adiposity impacts upon cutaneous perfusion dynamics. Methods: Mild cool challenge, followed by real-time video thermography, was developed to map the appearance of abdominal ‘hot spots’. Correspondence of marked ‘spots’ with audible Doppler and colour and power Doppler ultrasound was performed. Results: 60 healthy, afebrile, women (20–68 years; BMI 18.5–44 kg/m^2^) were recruited. Hot spot appearance consistently corresponded with audible Doppler sounds. Colour and power Doppler ultrasound revealed vessels at depths of 3–22 mm. No statistically significant interactions for BMI, abdominal circumference or environmental parameters were observed for hot spot count. The temperature of cold stimulus was significant for effects on spot count, but only for the first minute (*p* = 0.001). Thereafter, effects on spot numbers were not significant. Conclusions: Cutaneous ‘perforator’ mapping of the abdomen (via hot spot appearance) in healthy women, as a potential and future method for risk of perfusion-dependent wound healing complications, reveals that bedside mapping of skin perfusion is feasible over a short interval. Hot spot number was not influenced by BMI or indicators of central fat distribution (abdominal circumference) indicating variability in an individual’s vascular anatomy. This study provides the underpinning methodology for personalised perfusion assessment after incisional surgery which may be a more reliable indicator of potential healing complications than body habitus as is currently the norm.

## 1. Introduction

As a major surgical procedure in women of child-bearing years [1], caesarean section (CS) is not without complications [2]. In the post-operative period, slow healing and wound infection increases morbidity [3] and in rare cases, life-threatening infections [4]. One of the major indications for CS birth is obesity [5], a health risk that also increases the incidence of infection at the incision site; surgical site infection (SSI) [6,7].

Of the factors that predispose one to SSI, recent studies report an association with temperature. Differences in the thermal map, produced using infrared thermography (IRT) of incisional wound, during the first days and weeks after CS, showed temperature differences between healthy and wound sites exceeding 2 °C in women with established wound infection [8]. More specifically, it was low temperature ‘cold spots’ along and around the wound that emerged as feature signatures of later wound infection. Subsequent studies [6] confirmed a significant association between low abdominal temperature and wound outcomes; the lower the temperature the higher the odds of SSI. For example, low abdominal temperature at post-operative day 2 made a substantive, and by day 7 a significant (three-fold), contribution to increased risk of SSI. As shown by Savastano [9], the high fat content of adiposity and increase in tissue insulation results in lower abdominal skin temperatures compared with normal weight individuals, a feature also observed by Siah and Childs [10] in a healthy SE Asian population of participants.

In health, changes in skin temperature are primarily dependent upon oscillations in skin blood flow adjusting cutaneous perfusion [11]. Under the control of the efferent limb of the thermoregulatory centre, variation in skin temperature is due to heat flux from body core to the environment. In effect, skin perfusion, and thus temperature, is seldom uniform. When visualised using specialised thermal cameras, skin appears as a thermal ‘mosaic’ [12]. 

The interplay between skin perfusion and skin temperature can also be a direct consequence of systemic disease. In critically ill patients, for example, the effect of circulatory shock can result in cutaneous hypoperfusion, especially at the extremities, worsening healing rates in pre-existing chronic wounds and ulcers [13]. Here, evidence for the effect of poor skin perfusion and concomitant effects on skin integrity can be seen in the most severely ill where perfusion ‘deprivation’, indirectly measured by mean arterial pressure (MAP of <80 mmHg), is linked to wound deterioration [14]. The underpinning dependence of wound healing on cutaneous perfusion reinforces the significance of poor skin perfusion in prolonged or stalled wound healing [15]. Whilst MAP is a broad and useful guide to risk of poor healing in clinical practice, alternative techniques exist which measure the physiological state of tissue attributed to blood flow [15]. 

In view of concerns and vulnerability of obese patients to surgical site infection (SSI) [16,17] more information is warranted on the relationship between adiposity, skin temperature and perfusion in obese people undergoing surgery because recovery occurs only on complete healing of the skin and wound. To achieve this, skin must be viable. It must receive an optimum supply of blood and nutrients and this, in health, is achieved by perfusion of blood to skin via abundant vascular networks arising from deep source vessels, their angiosome territories [18], perforators and branches which supply the subdermal-plexus. 

In the context of lower abdominal surgery, cutaneous perfusion in the region of a Pfannenstiel incision [19] is via source vessels of deep systems, primarily deep inferior epigastric artery (DIEA) and its perforating branches [18,20]. Perforators of DIEA course through rectus musculature and overlying fascia to reach the subdermal plexus, the main supply to the skin [21]. Abdominal skin blood supply is also from the superficial inferior epigastric artery (SIEA) [22]. Arising from the femoral artery, approximately 1 cm below the inguinal ligament, the SIEA travels superficially and superiorly between abdominal fascia towards the umbilicus [23]. Unlike the DIEA, the SIEA is not ‘classically’ considered a perforator, due to a lack of uniformity of branches lying deep to Scarpa’s fascia [24]. However, SIEA does have a deep origin and at some point, one or more of its branches pierce Scarpa’s fascia [25] to follow a fascio-cutaneous course. Thus, in studying cutaneous perfusion, both deep and superficial systems play a role in influencing cutaneous perfusion to lower abdominal skin regions and ultimately temperature patterns across the abdomen. 

The objective of this study was to explore whether a relationship exists between BMI, and cutaneous perfusion by mapping the number of abdominal perforators. Dynamic infrared thermography (DIRT) was the method under test in response to mild cold challenge. The first step, undertaken in healthy women, was to test feasibility and acceptability of the method of induction of thermal challenge and to verify perforator location as evidenced by cutaneous ‘hot spots’ on DIRT and to verify DIRT methods against three independent, non-invasive imaging modalities; hand-held vascular Doppler, power and colour Doppler. 

## 2. Materials and Methods

### 2.1. Study Design

A prospective imaging methods development study with intervention feasibility was conducted in three phases over a period of 10 months. Phase 1, detector calibration and cold challenge method testing; phase II, perforator location and number across different BMI categories using DIRT, phase III, correspondence and validation of DIRT identified cutaneous perfusion (hot spot location) with independent measures of skin perfusion/blood flow.

Sheffield Hallam University institutional ethical approval for studies on human participants was obtained before research on healthy subjects commenced. All equipment used was safety checked and risk assessed. All investigators and participants followed NHS COVID-19 guidelines for infection prevention and control.

### 2.2. Phase I: IR Detector Calibration

**Figure 1:**
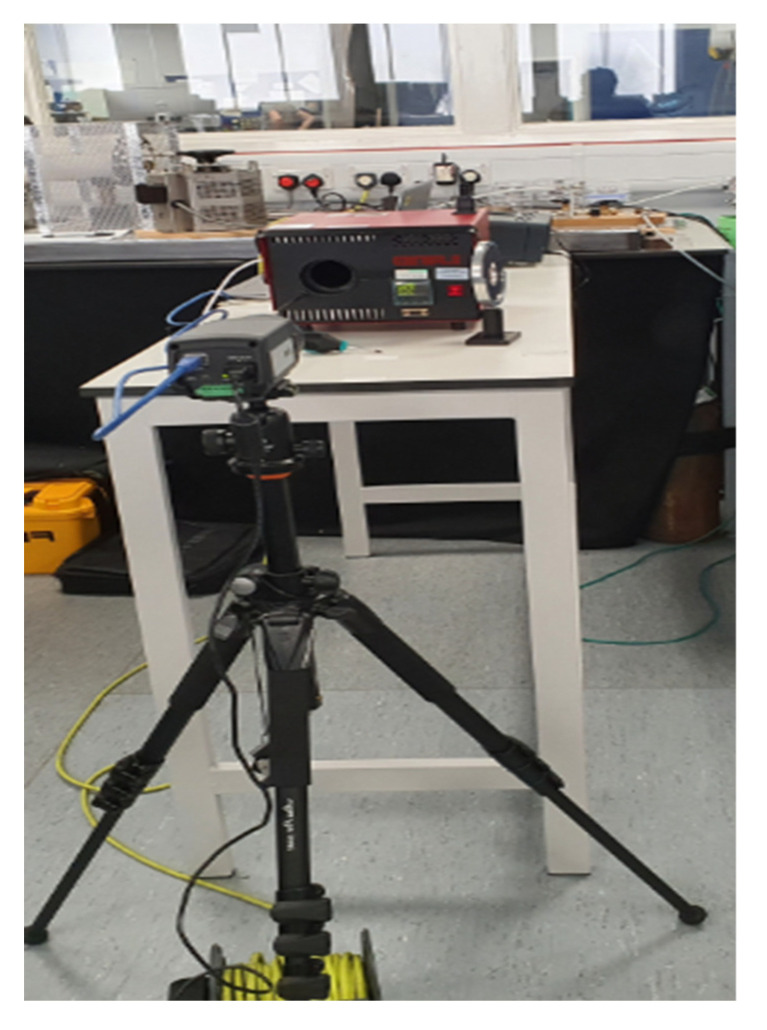
Calibration set up. IR detector mounted on tripod with lens directed to Black body radiator.

Thermography was undertaken using a FLIR Systems A655sc (640 × 480 pixel resolution), uncooled, science grade, microbolometer with 25° lens and with thermal sensitivity <30 mK. Images and data analysis were via ethernet streaming (30 Hz) to a laptop PC running FLIR Research IR Max software V4.40.11.35 (64 bit). The IR detector was calibrated, before study start, and at mid-recruitment point, using a black body source (Ametek-Land, Dronfield, UK) (Figure 1). IR detector readings were compared to a certified (UKAS, UK) independent type 100 Ω platinum thermometer (PRT100, ISOTECH, Skelmersdale, UK) placed in situ within the black body. Over a period of 4 h, and at an incremental temperature span of 10 °C (27.5 °C to 37.5 °C), the difference between IR detector readings and PRT ranged from 0.20 to 0.5 °C (median 0.41 °C).

### 2.3. Phase II

#### Selecting a Suitable Method of Mild Thermal (Cold) Challenge

Six different methods were tested for their performance and acceptability to lower skin temperature within a period of 2–5 min; metal floor standing fan, plastic lightweight fan, large ‘Magic Gel’™ cold compress (38 × 28 cm), wet wipes (alcohol containing/no alcohol), operating table torso pad (Anetic Aid, Baildon West Yorkshire, UK) and plastic (with sealable tap) inner packaging of a 10 Litre ‘bag in box’ system (Vigo Ltd., Honiton, Devon, UK) filled with 5.4 L of tap water. With the exception of the plastic insert from Vigo’s ‘bag in a box’, none of the methods tested, be it forced convection (fan), local conduction (gel pads), latent heat of vaporization (wet wipes) proved practical or reliable in lowering skin temperature for more than a few seconds. Skin rewarmed rapidly on removal of the material. By contrast, the sealed tap water-filled plastic container from the ‘Bag in a Box’ packaging proved robust, leakproof and maintained a consistent water temperature, sufficient to lower skin temperature by approximately 10 °C. In the summer months the bag was placed in a larder refrigerator for not more than 15 min to lower water temperature before use.

### 2.4. Phase III

#### Thermal Challenge and Dynamic Infrared Thermography Mapping of Cutaneous ‘Perforators’

**Figure 2:**
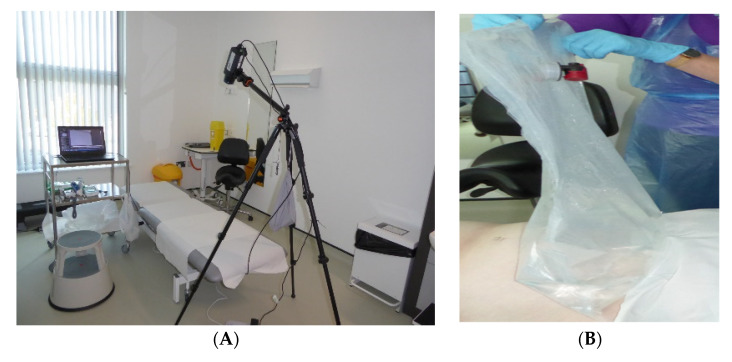
Clinical set up, (**A**) Tripod mounted thermal imaging detector positioned over examination couch for Field of View to include abdomen. (**B**) Water-filled bag at 18–20 °C applied to abdomen to induce local thermal challenge.

Environmental monitoring: Ambient conditions for air temperature (°C), relative humidity (RH%) and air velocity (m·s^−1^) were recorded every 10 min with a weather meter (Kestrel 3000, Richard Paul Russell Ltd., Hampshire UK) positioned at the site of the tripod. The infrared detector, mounted on a tripod at 45–50 degrees to the abdomen, was stabilised using a 1 Kg weight suspended centrally (Figure 2) and connected via ethernet cable to a laptop personal computer. The tripod legs were aligned to ensure that the thermal detector was positioned towards the abdomen to provide a central field of view (FOV).

Protocol for DIRT and perforator mapping of healthy women: Participants: Healthy women over the age of 18 years were eligible for recruitment irrespective of parity (nulliparous women were eligible) or past mode of delivery (vaginal birth or caesarean section). Participants were recruited via study posters, social media, word of mouth and networking. Purposive sampling, with a target of 60 participants, was chosen to reflect a cross-section of healthy women across three BMI categories, healthy (normal) weight (18.5–24.9 kg/m^2^), overweight (25–29.9 kg/m^2^) and obese (≥30 kg/m^2^) (https://cks.nice.org.uk/topics/obesity/diagnosis/identification-classification/, 14 February 2023).

Women attended University equipped clinical consultation rooms under standard ambient conditions during the period from November 2021 to July 2022. Air conditioning was not used. On arrival, study activities were reviewed and signed informed consent obtained. Demographic information, obstetric history and pre-existing medical conditions were documented along with body temperature measured at the tympanum (Thermoscan, (Model LF 40, Braun, Lausanne, Switzerland), weight, height and abdominal circumference at the level of the umbilicus. Ambient conditions, including temperature, relative humidity (RH%) and air velocity (m·s^−1^) were recorded throughout the 50 min of study. Lights were turned off and blinds closed.

Lying supine, the abdomen was exposed, from the level of the umbilicus to the bikini line, for a period of approximately 5 min whereby women were reassured of the cool challenge procedure and allowed to settle and feel comfortable. One sheet was placed to cover the upper abdomen and chest and a second to cover the pubic region at the level of the groin. This was followed by a further 10 min of timed cooling. The objective was to achieve environmental acclimation following guidelines of the International Association of Certified Thermographers (2020) (https://iactthermography.org/standards/medical-infrared-imaging/, 14 February 2023). Subjects were requested to lie still throughout, with arms and hands at their sides or over the chest.

**Figure 3:**
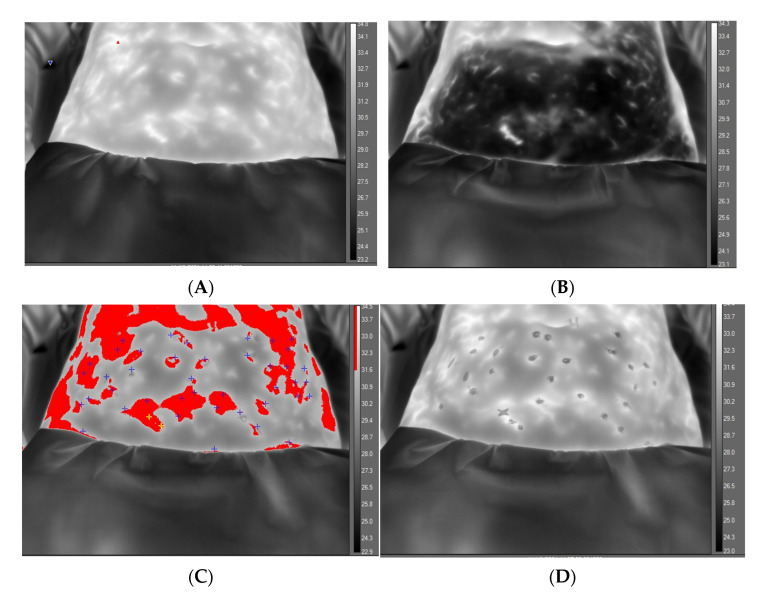
Greyscale thermal image of abdomen at baseline, before application of cold challenge and with temperature scale, (**A**). Abdomen appearance after 2 min of cooling showing effect of cooling in reducing regional temperature, (**B**). Abdomen appearance at 10 min, revealing ‘hot spots’ distinguished by isotherm feature (red) and with post-processing markers (+) indicating location of perforators, (**C**). Final video-thermography frame at end of recovery phase with isotherm removed and ‘spots’ indicating location of marked hotspot before perforasome evolution, (**D**).

IR image capture: IR parameter settings: Emissivity was set to 0.98, distance to 60 cm, with reflective ambient temperature of 20 °C and ‘greyscale’ image palette selected. At the end of 15 min acclimation period (5 min + 10 min), a digital image of the abdomen was taken, along with baseline static IR image (Figure 3). A ROI was drawn to cover the central abdominal area from which data to obtain mean, maximum and minimum temperature vales within the boundary was acquired.

From the ROI data, an isotherm feature (selected red to contrast within the greyscale image) was set (a span of 3 °C below the highest measured temperature within the ROI before cooling). This feature makes for better contrast and identification of hot spots (temperature recovery) as they appear. Video recording was set to 10 min at 30 Hz and commenced immediately on removal of cold challenge.

Cold Challenge: Two minutes of mild cold challenge was applied to the lower abdomen, with tap water temperature at 18–20 °C. The bag was ‘rolled’ gently over the abdomen, redistributing water to achieve a uniform temperature reduction. Immediately upon removal, video recording commenced. After removal of cold challenge, a lowering of skin temperature was apparent (Figure 3B). The appearance of a ‘hot spot’ appeared against the greyscale background and location was marked on the skin with metallic, non-permanent, marker pens. On completion of the video, recordings were saved for post-processing. Ultrasound gel was then applied over all marked spot locations and a hand-held Doppler probe (8 Mz) (Huntleigh, Cardiff UK) applied to listen for arterial sounds and to view the concomitant waveform (CC and HN).

### 2.5. Correspondence between DIRT Identified ‘Perforators’, Vascular Doppler and Colour and Power Doppler

**Figure 4:**
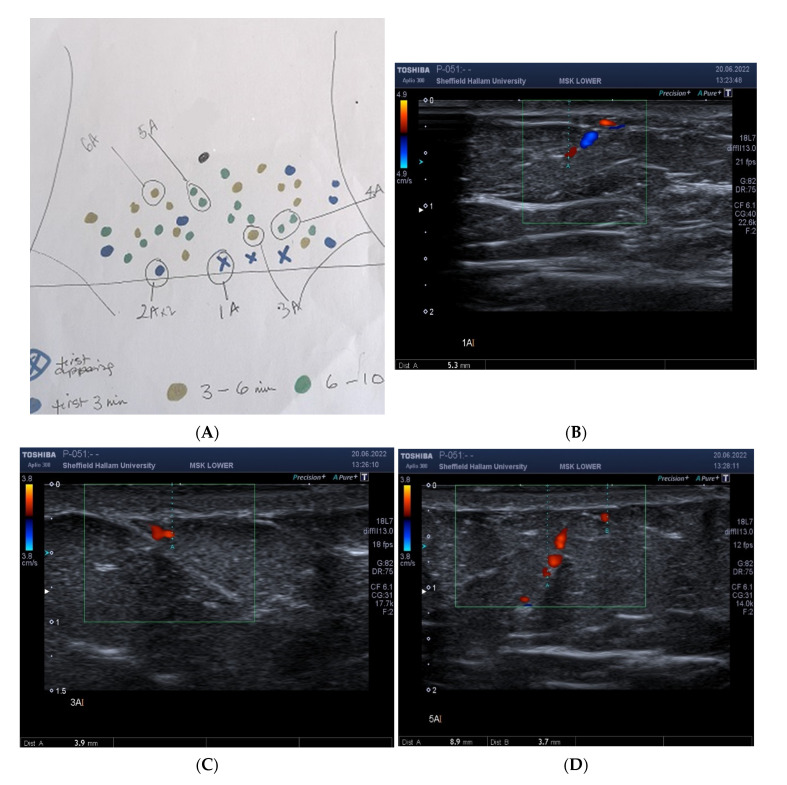
Correspondence of hot spot locations to guide position of Doppler ultrasound probe. Location of abdominal hotspots as they appeared in real-time, and mapped manually, as a sketch at the bedside, during 10 min video thermography (**A**). The labels were drawn to identify the sequence of appearance and to guide the sonographer for placement of the ultrasound probe. Blue marker (X) indicates first spot/s to appear. Blue circles, hot spot appearance within the first 3 min; gold, 3–6 min; green, 6–10 min, A. In this subject, three of the first hot spots appeared simultaneously and were marked accordingly by blue (X). Depth of vessel (5.3 mm) at hotspot (1A) measured using colour and power Doppler Ultrasound, (**B**). Vessel depth at 3 A 3.9 mm, (**C**). Depth of vessels at spots 5 A, 8.9 mm and 3.7 mm, respectively, (**D**).

In a sub-group of participants, an in-depth Doppler ultrasound examination (Aplio 300, Toshiba Systems, CA, USA) was performed by an experienced sonographer (EB). Participants were positioned supine, and with an empty urinary bladder, on an ultrasound scanning couch. A 12 MHz linear probe was used to perform the ultrasound examination, using a pre-set programme of ultrasound settings for consistency and reproducibility of parameters (gain and dynamic range). A starting depth from a skin surface of 20 mm was set. Both colour Doppler and power Doppler were used to assess for vessel presence. A colour box was positioned over the existing gel covered marked hotspot. Colour Doppler imaging was used with the advantage of detecting direction of blood flow [26]. The colour scale was set between 3.8 cm/s and 4.9 cm/s. Power Doppler was also used for its reported higher sensitivity to flow with better edge definition compared to colour Doppler imaging [27]. Each identified vessel was measured with the caliper measurement tool placed at the deepest part of the vessel from the skin surface (Figure 4). Each ultrasound scan was labelled to correspond with the marked spot identified on the thermal image. Data were stored on a secure University network drive.

### 2.6. Statistical Analysis

The relationship between the independent variables (age, BMI, ambient temperature, and bag temperature) and the outcome variable (spot count) was investigated over the first 5 min of DIRT recording using Poisson generalised linear mixed effects regression models. A separate model was fit for each of the independent variables with time included as a fixed factor and participant ID included as a random intercept. An interaction term between the fixed factor for time and the independent variable of interest was included to test if the relationship with the outcome changed over time. Negative binomial generalised linear mixed effect regression models were also considered but after inclusion of the random effect for participant ID there was no evidence of overdispersion, and the negative binomial models failed to converge. For each time point the relationship between the independent variables and outcome variable is described as an incidence rate ratio (IRR) per one unit increase in the independent variable (e.g., a one unit increase in BMI) along with a 95% confidence interval (CI) and *p*-Value. All analysis was conducted using the statistical analysis software R (4.2.1) (https:www.R-project.org/, 14 February 2023).

## 3. Results

A total of 60 afebrile healthy women, aged 20–68 (mean 42.3) years, of diverse ethnicity were studied. Abdominal circumference ranged from 65–147 (mean 91.3) cm, body mass index (BMI) 18.5–44.0 (mean 27.4) kg/m^2^. Over one third (37%) were obese; 50% overweight/obese (Table 1). None of the participants had cardiovascular disease. Two women had type II diabetes and two women smoked. Of 40 parous women recruited, 16 (42%) had given birth by C-section. A total of 15 women (25%) were either menopausal or post-menopausal (Table 1) at the time of study.

**Figure 5:**
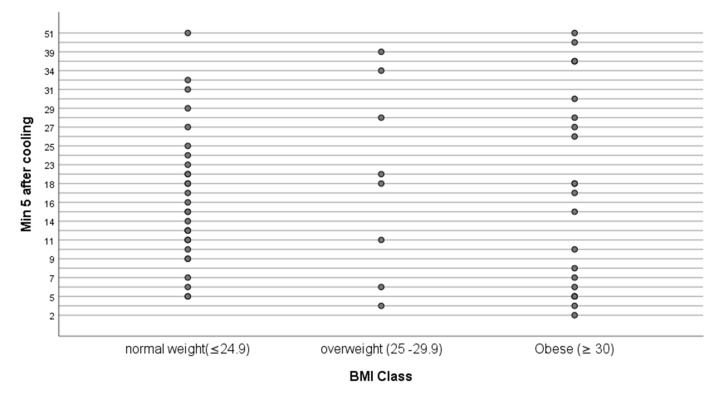
Hot spot count at 5 min after the end of cooling by BMI Class. BMI, kg/m^2^.

The environmental temperature of the clinical room was relatively constant during winter to summer months of the study (Table 2). After 15 min acclimatisation, the exposed abdominal temperature ranged from 29.8–34.7 °C. Application of cold challenge (via water cooled plastic ‘bag’) reduced skin temperature by approximately 5–10 °C over a duration of 90–180 min (n = 45 women cooled for 120 s; n = 12 cooled for 90 s, n = 3 cooled for 180 s). During the first 5 min of the 10 min DIRT video recordings, the number of marked hot spots totalled 1112, individual count ranging from 2–51 (mean 18) spots (Figure 5). Hot spot count for 60 participants at the 1 min timepoint: 0–16 (mean 2.8, SD 3.4); at 2 min, 0–26 (mean 6.3, SD 5.7); at 3 min, 1–42 (mean 10.8, 8SD 8.6); at 4 min, 1–51 (mean 14.9 SD 10.5) and at 5 min, 2–51 (mean 18.5 SD 12.0).

The major concerns for the methodological approach to thermal challenge were in the interaction between time and independent variables that could influence skin perfusion, as evidenced by hot spot count on DIRT. There was not a statistically significant (*p* = 0.122) interaction between time and age indicating that the relationship between spot count and age does not change over time. The relationships at each time point were not statistically significant and the incidence rate ratios (IRR) were close to 1 suggesting that there is no real evidence of a relationship between age and spot count.

With respect to BMI, the interaction between BMI and age was not statistically significant (*p* = 0.954) indicating that the relationship between spot count and BMI does not change over time. The relationships at each time point were not statistically significant and the IRRs were close to 1 suggesting that there is no real evidence of a relationship between BMI and spot count.

The interaction between BMI and ambient temperature was not statistically significant (*p* = 0.913) indicating that the relationship between spot count and BMI does not change over time. The relationships at each time point are not statistically significant and again the IRRs are close to 1 suggesting that there is no real evidence of a relationship between ambient temperature and spot count.

The interaction between time and bag temperature was statistically significant (*p* = 0.001) indicating that the relationship between bag temperature and spot count changes over time. At 1 min there was a statistically significant relationship (IRR: 1.240, 95% CI: 1.014 to 1.516, *p*: 0.036) showing that the spot count rate increases by 24% for each 1 °C increase in bag temperature. However, at 2 min (IRR: 1.101, 95% CI: 0.921 to 1.218, *p* = 0.291) and 3 min (IRR: 1.042, 95% CI: 0.878 to 1.26, *p* = 0.640) the rate of increase decreased to 10% and 4%, respectively, but these relationships were no longer statistically significant. At 4 min (IRR: 0.983, 95% CI: 0.831 to 1.162, *p* = 0.839) and 5 min (IRR: 0.971, 95% CI: 0.822 to 1.146, *p* = 0.730) the point estimates were less than 1 suggesting a decrease in the spot count rate, but the relationships were not statistically significant.

### Correspondence between DIRT Located Hotspots and Independent Measures of Skin Perfusion

**Figure 6:**
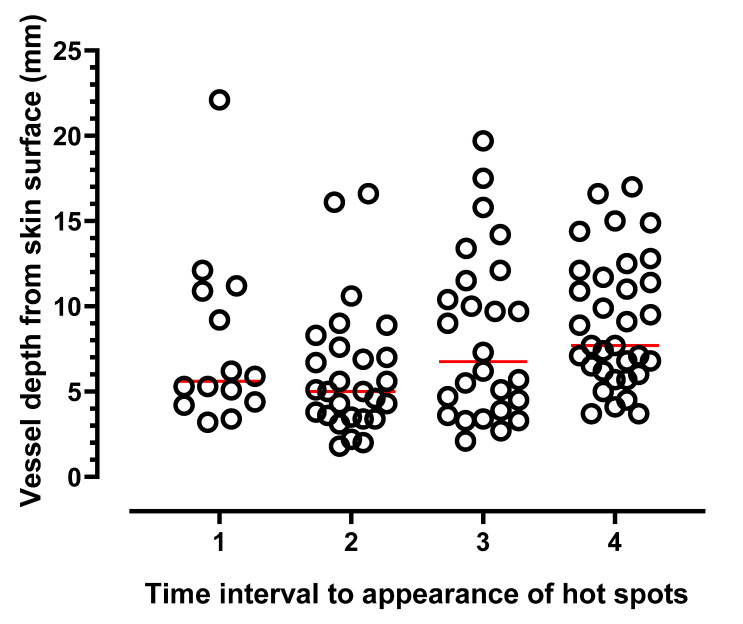
Depth of vessels in 14 participants measured at hot spot locations using ultrasound calipers. Measurements taken from skin surface to vessel base. First appearing hot spots, (time 1). Thereafter, a further 5–6 spots in each colour category (blue, gold, green) were selected from the map (see Figure 4A); T2, vessel depth of spot/s appearing within 3 min; >3–6 min (T3); spot appearance >6–10 min (T4). Horizontal lines indicate median values.

Of the 60 women studied, audible arterial Doppler sounds were heard at the majority (98%) of marked hot spots during the 10 min DIRT test. For the last 14 participants, Doppler ultrasound (colour and power mode) was used to confirm vessel presence (Figure 4). At first spot appearance vessel depth from skin surface ranged from 3.2–22.1 (median 5.3) mm (T1, Figure 6). For subsequent spot appearances, arbitrarily divided to three periods; (a) within 3 min (T2); (b) >3 min to 6 min; (T3) (c) >6 min to 10 min (T4), vessels corresponding to hot spots appearing 6–10 min after removal of cold challenge, were significantly deeper than those arising within the first 3 min (*p* = 0.003).

## 4. Discussion

Perforator mapping using DIRT techniques has been undertaken extensively in studies where the focus is on flap harvest as well as for monitoring of microvascular insufficiency after flap surgery [28].

Perhaps the most compelling evidence for the use of DIRT has been as a surrogate for blood perfusion in the selection of the most suitable perforator for breast reconstruction with an autologous free flap of the abdomen. Here, knowledge of perforator location and skin perfusion is a key element of successful autologous flap harvest [29,30,31,32,33,34] as well as intraoperative and post-operative monitoring of flap viability. In the context of wound healing problems, especially following abdominoplasty, complications at the lower transverse suture line is a major risk for delayed wound healing linked to poor perfusion consequent upon the undermining of skin and subcutaneous tissue to produce a large skin flap [35]. Extending the role of IRT across surgical specialties, and as a relatively inexpensive, non-invasive technique, interest in its potential to identify microvascular insufficiency has applications in vascular surgery and flow-related obstructive peripheral vascular disease [36] diabetic foot [37,38], and critical limb ischaemia [39].

To our knowledge, as a proof of concept, this is the first study to specifically map abdominal perforator number and location in healthy women using DIRT video imaging.

In addition, we have shown the impact of cooling on surface temperature. A reduction in skin temperature of just 8–10 °C is sufficient to allow skin perfusion to provide an excellent endogenous contrast such that the recovery of temperature (as hot spots) is clearly visible to the observer. This is a major advantage for the practical application of DIRT in medicine. The results suggest that DIRT has potential applications for the future as a clinically feasible intervention for post-operative skin and wound perfusion evaluation after CS.

Of the physiological factors known to influence skin temperature, the most widely appreciated with potential diagnostic utility in clinical medicine is inflammation and ischaemia. Both are accompanied by changes in local cutaneous blood perfusion and temperature [40]. For example, conditions which lead to high cutaneous blood flow and emitted IR heat include inflammatory skin lesions [41] and the peri-wound regions of chronic ulcers [42]. By contrast, perfusion deficit leading to low skin temperature is a feature of ischaemic wounds [43].

Thermography is usually undertaken in either static or dynamic modes. Although static IRT provides useful information to map skin temperature under ‘steady state’ conditions, it fails to show changes in temperature and, by proxy, perfusion, over time. However, in dynamic mode, IRT provides an indirect technique for monitoring of vascular perfusion following an applied thermal challenge. To achieve a ‘dynamic’ state, a short period whereby an induced thermal challenge produces a modest change (fall or rise) in skin temperature is required. In most circumstances, skin cooling is instigated either by convection [32] evaporation [44,45] or conduction [46,47].

On removal of the challenge, thermal recovery is marked by the appearance of ‘hot spots’; the first ‘spot’ indicating the return of ‘warm’ blood to the skin. This feature of dynamic (or ‘recovery enhanced’ [48]) thermography has been used in flap surgery as an adjunct imaging modality alone, or in conjunction with traditional radiological techniques to locate ‘dominant’ perforators and their branches as they reach subcutaneous tissue. In the planning and monitoring of fascio-cutaneous flaps, DIRT provides a reliable technique for perforator selection [30], a method comparable to ‘gold standard’ computed tomography (CT) angiography [32]. Other imaging modalities also used to confirm DIRT reliability in identifying perfusion include hand-held Doppler ultrasound alone [30] or in combination with CT angiography [34] and indocyanine green (ICG) fluorescence video angiography [49,50,51]. Although several drawbacks exist in the use of conventional radiological techniques with respect to safety (radiation exposure), high cost and user skill, it is clear when comparisons are made between DIRT and the established methods of perfusion dynamics, that DIRT performs well in the early detection of perfusion deficits due to tissue ischaemia. However, little is known of the potential for DIRT as an adjunct to assessment and prognosis of wound healing complications due to stalled healing, infection or ischaemia consequent upon altered skin perfusion, especially in high-risk patients with pre-existing co-morbidities.

In this study of healthy women, and using cold water challenge, skin temperature was lowered by an average of 8 °C over a period of 2 min; in essence a moderate stimulus which avoids discomfort. Alongside the cold challenge, dynamic thermography enhances image contrast. In essence, recovery of warm blood to the skin acts as an endogenous contrast ‘agent’. In this study, return of warm blood was observed on video thermography as a pulsating pixel ‘hot spot’, standing out against the cooled skin background. The technique allowed for immediate marking of each ‘spot’ as it appeared and evolved (as a gradually expanding halo around the first appearing pixels) which eventually join up with adjacent hot spots to produce a perforasome [52]; the vascular territory supplied by a single arterial perforator [53].

Throughout the first 5 min of video thermography, a range of hot spot appearances were evident; from as little as 2 to a maximum of 51 (median 16) and with 5 hotspots the most frequent number.

By 10 min, the thermal map appearance largely returned to the pre-cooling thermal state; individual hot spots were no longer identifiable, replaced by a near confluent temperature distribution, close to that observed at baseline. It has also been possible to demonstrate that as each hot spot emerged, verification by hand-held Doppler showed that 98% of the marked spots corresponded to an audible arterial sound. Furthermore, confirmation that a hot spot location reflected the site at which underlying direct and indirect [52] vessels were present was shown from the scans obtained using colour and power Doppler ultrasound. This is consistent with reports of Xiao et al. [54] when comparing IRT with colour Doppler. Hot spot appearance on IRT had a high degree of consistency. However, whilst hot spots mark the point at which perforators enter the skin- the ‘terminal’ entry point may be direct (travelling vertically) or indirect (travelling horizontally) after piercing the deep fascia. Xiao et al. [54] have shown that spot location on thermography when compared to colour Doppler can lead to some deviation in position due to thickness of subcutaneous tissue. Whilst Xiao et al. [54] suggest that IRT is particularly useful to detect hot spot location in flap design for those with thin subcutaneous tissue, our experience, at least in terms of using hand-held Doppler to verify vessel presence, was that sounds were heard at marked locations in those with a range of subcutaneous fat thickness as evidenced by BMI.

Despite the diversity in BMI of participating women in this study, being overweight or obese (reflected as BMI and abdominal circumference) did not influence hot spot number. This may be due to the limited number of participants at each BMI category, but it may also suggest that vascular perfusion and heat distribution across the skin may be influenced more by vascular anatomy than by thickness of subcutaneous fat depots. For example, 5 min after removal of cold stimulus, the number of marked hot spots in three women with the highest BMI (44.0, 41.74 and 42.76 m^2^/kg) numbered 30, 6 and 5, respectively, indicating anatomical variability in the number of perforators as they arise from deeper vessels towards the skin surface; a finding reported previously by a number of authors examining the vascular supply of the anterior abdominal wall [19,52,55,56].

For the 15 women who had previously undergone C-section surgery, dissection of adipocutaneous tissue and underlying muscle fascia might be expected to impact on skin perfusion. With the average length of time since surgery (with the exception of one woman) being 8 years (2–19 years) no clear distinction regarding the appearance of hot spots along the vestigial scar was observed.. However, it should be noted that a number of women did report a lack of sensation in the region of the scar suggesting long lasting effects of dissection on skin sensation.

Confirmation of vessel depth at the hot spot sites was made using colour and power Doppler ultrasound. Vessel presence and flow direction was observed at each tested hot spot location, albeit with varying depths from skin surface. Whilst hot spot count *per se* appears unrelated to subcutaneous adiposity (BMI and abdominal circumference), in the absence of skin fold thickness it is, as yet, unclear whether vessel depth influences the speed at which hot spots appear. However, there is some evidence from the data that first appearing hot spots tended to be closer to the skin surface than those hot spots that emerged later. Therefore, whilst subcutaneous thickness did not appear to influence hot spot count, it may have a relationship with speed of recovery of hot spots.

As a potential method for future SSI risk stratification of perfusion-dependent wound healing complications, the method is feasible and uncomplicated but further confirmatory work needs to be undertaken with respect to consistency in achieving the desired cold challenge temperature (in this study, the temperature of the water-filled bag) as well as translating the technique from healthy participants to surgical patients. From this methods development study it is possible that, in addition to external factors influencing wound healing, there may be an inherent, individual (anatomical) susceptibility to poor wound healing that could be explained by an individual’s vascular anatomy and ultimately the ‘vitality’ of skin perfusion given the disruption of vascular networks consequent on vessel dissection and tearing during surgery. Here, post-operative perforator mapping has potential as an objective, personalised, medicine strategy, easily tested, in real-time, at the bedside as an indicator of the potential perfusion state of the cutaneous microvasculature in the region of a planned incision. Whether a reduction in perforator number or speed of recovery after moderate cold challenge could be a ‘flag’ for later post-operative wound healing complications remains unclear but from our previous studies, we know that the appearance of cold spots along the wound site marks, in many cases, a prodrome for later SSI. The appearance of low temperature ‘cold spots’ (within a well perfused wound area) likely reflects avascular (or poorly perfused) regions which ultimately influence the skin heat map [57]. Whilst many pre- and post-operative factors, both internal (smoking, immunity, obesity) and external (asepsis, surgical technique, surgeon skill, emergency CS) are associated with SSI risk [58], it is recognised that wounds will not heal if tissue perfusion is inadequate [14]. Thus, knowledge of cutaneous perfusion during the peri-operative period has promise as a method for skin (and wound) perfusion deficits. Whilst there is some evidence, at least from studies in free flap surgery, particularly the SIEA flap, that a reduction in perforator number carries a higher risk of fat necrosis [59], it is not yet possible to tell from results of this study whether differences in the number of perforators supplying skin in the region of the surgical incision has a bearing on perfusion-dependent wound healing outcomes.

Finding a method to induce mild cold challenge and subsequent hot spot mapping has promise for future investigations even though some difficulties still need to be resolved, not least in controlling the temperature of the applied stimulus. Whilst a similar, water filled, bag system has been used successfully for intra-operative use [47], the method ultimately lacks a reliable means to adjust water temperature. In this study, bag water temperature was maintained from 18–22 °C across the seasons, a range sufficient to lower abdominal skin temperature effectively by 8–10 °C. Our concern was the relationship between bag temperature and influence on skin temperature recovery marked by hot spot appearance. Control of the temperature of the applied stimulus is warranted to improve consistency. However, for this study at least, after 1 min of recovery, spot count was not significantly influenced by the initial temperature of the bag applied to skin. This gives a benchmark for the timing to thermal recovery, i.e., that the duration to observe for hot spot appearance should be more than one minute. Indeed, whilst a lower bag temperature increases image contrast between hot spot and surrounding skin, it does not alter the location or rate of recovery of the hot spot which defines the anatomical site of the perforator. For pragmatic purposes, 5 min provides a realistic rewarming phase to conclude the thermal recovery period for perforator location and evaluation.

## 5. Conclusions

In everyday healthcare objective wound and skin edge imaging is seldom done; clinicians rely more on experience and visual assessment and/or the use of any number of wound assessment tools available to them in their practice. What cannot be seen by eye is missed. However, objective, independent IR imaging technology could help clinicians assess whether avascular regions exist along or around the wound. This would provide an early signature of incipient tissue damage which may ultimately lead to delayed healing or even to a surgical site infection prodrome. Perforator mapping using DIRT could be a potentially valuable tool for stratification of high-risk patients in evidence-based antibiotic prophylaxis.

In this study a new approach to skin perfusion assessment was tested in healthy participants using dynamic long-wave infrared video thermography before and after 2 min of a mild cold challenge to abdominal skin. The technique was shown to be feasible over a period of 10 min but for practical purposes perfusion-dependent thermal mapping is achievable and robust over a shorter interval of 5 min. In this study, hot spots marked the position of cutaneous perforators, but number of hot spots was not significantly influenced by adiposity indicating that it is individual variability in the number (and thus position) of perforator vessels rather than subcutaneous adiposity that most influences the dynamics of the abdominal thermal map. For future applications in surgical patients, results of this study may help to shape future approaches to wound complications risk assessment by emphasising the clinical importance of personalised cutaneous perfusion mapping rather than risk stratification by body habitus as is currently the norm.

## Figures and Tables

**Table 1:** Participant Characteristics.

Factor/Parameter	Frequency (Valid %)	Range	Mean (SD)
**Age (years)**		20–68	42.3 (10.8)
**Ethnicity**			
Arab	2 (3)		
Asian	2 (3)		
Pakistani	2 (3)		
Persian	1 (2)		
Black: African	1 (2)		
Black: British	1 (2)		
Latino	1 (2)		
White British	43 (72)		
White Other	7 (12)		
Height (cm)		149.5–178.8	165.0 (7.0)
Weight		49.4–115.0	74.7 (17.9)
Abdominal Circumference *		65.0–147.0	91.3 (18.7)
BMI Kg/m^2^		18.52–44.0	27.4 (6.6)
**BMI Kg/m^2^**			
Healthy weight (≤24.9)	30 (50.0)		
Overweight (25–29)	8 (13.3)		
Obese (≥30)	22 (36.7)		
**Parity**			
Parous	40 (33.3)		
Nulliparous	20 (66.7)		
Number of women who had a C-section birth	16 (42%)		
**Co-morbid conditions**			
CVD **	0		
Diabetes	2 (3.3)		
Smokes	2 (3.3)		

* measured at the level of the umbilicus, ** CVD, Cardiovascular Disease.

**Table 2:** Environmental conditions, body and skin temperature variables before and after thermal challenge.

	N=	Range	Mean	SD
**Ambient conditions at study start**				
Air temperature (°C)	60	18.3–23.8	21.3	1.5
Relative Humidity (RH%)	60	30.3–69.3	45.2	6.5
Air velocity (m·s^−1^)	60		0–0	still air
**Tympanic temperature (°C)**	60	35.8–36.5	36.8	0.3
**ROI: Abdominal skin temperature before cooling (°C)**				
ROI max	60	31.8–36.2	33.8	0.90
ROI mean	42	29.8–34.7	31.9	1.13
**3 °C isotherm: minimum to maximum temperature (°C) ^†^**	60	28.8–33.2	30.8	0.93
**Bag temperature at start of cooling (°C)**	60	17.8–22.0	19.4	1.2
**Abdominal skin ROI temperature after cooling**				
ROI mean (°C)	60	22.0–27.5	24.3	1.1
** Temperature difference: before and after cooling: maximum abdominal skin temperature (°C)	60	6.8–11.7	9.5	1.2
*** Temperature difference before and after cooling: **abdominal ROI (°C)**	42	4.8–10.6	7.9	1.2

**^†^** isotherm values within the 3 °C range appear as one (red) colour against grey scale thermal map (see Figure 3C). ROI, Region of Interest. ** calculation based on difference in maximum abdominal skin temperature before cooling minus ROI temperature at end of 2 min cooling. *** calculation based on difference in mean ROI temperature before cooling minus abdominal region of interest (ROI) temperature at end of 2 min cooling.

## Data Availability

The data presented in this study are available on request from the corresponding author. The data are not publicly available due to intellectual property restrictions.

## Creative Commons Statement

For the purpose of open access, the author has applied a Creative Commons Attribution (CC BY) licence to any Author Accepted Manuscript version arising from this submission.
